# Evaluation of Short-Term Changes in Fast-Food Restaurant Online Kids’ Meal Beverage Offerings Following a State-Level Healthy Beverage Default Policy

**DOI:** 10.1016/j.cdnut.2023.100045

**Published:** 2023-02-11

**Authors:** Lisa M. Powell, Julien Leider, Andrea A. Pipito, Alyssa Moran

**Affiliations:** 1 Health Policy and Administration, School of Public Health, University of Illinois Chicago, Chicago, IL; 2 Institute for Health Research and Policy, University of Illinois Chicago, Chicago, IL; 3 Health Policy and Management, Bloomberg School of Public Health, Johns Hopkins University, Baltimore, MD

**Keywords:** healthy default beverage, nutrition policy, kids’ meals, fast-food restaurants, childhood obesity prevention

## Abstract

**Background:**

Consumption of food and beverages from restaurants is associated with poorer diet quality and a higher intake of sugar-sweetened beverages (SSBs) among children, and SSBs are commonly offered as part of kids’ meals at restaurants. Thus, an increasing number of states and localities have mandated that only healthy beverages be provided by default with kids’ meals.

**Objectives:**

We examined changes in default beverages offered with kids’ meals 4 mo after an IL healthy beverage default (HBD) act took effect.

**Methods:**

A pre-post intervention-comparison site study design was used, with WI as the comparison site. Data were collected on default beverages offered on restaurant website or application menus at 64 restaurants in IL and 57 restaurants in WI in November 2021, before the IL HBD Act took effect, and May 2022, 4 mo after the date on which the Act took effect. Difference-in-differences weighted logistic regression models with robust standard errors clustered on restaurants were computed to examine changes over time in beverage offerings in IL relative to those in WI.

**Results:**

There was no statistically significant increase in compliance with the IL HBD Act’s criteria in restaurants in IL compared with those in WI (OR: 1.40; 95% CI: 0.45, 4.31). Although the compliance by fast-food restaurants increased from 15% to 38% in IL, there was a similar pattern in WI, with an increase from 20% to 39%. There were also no statistically significant changes in specific types of compliant beverages offered by default with kids’ meals in IL compared with those in WI.

**Conclusions:**

These results highlight the need for communication and enforcement to ensure that restaurants make changes in response to HBD policies broadly, including on their online platforms, and without substantial lags. Future studies should continue to measure the effectiveness of HBD policies alongside implementation strategies to determine how these policies can best achieve improvements in the nutritional quality of kids’ meals at restaurants.

## Introduction

Children and adolescents are frequent consumers of fast food. In 2015–2018, just over one third (36.3%) of the youth in the United States consumed fast food on a given day; and, 11.4% were heavy fast-food consumers on a given day, with >45% of their daily energy intake coming from fast food [1]. In 2017–2018, fast food accounted for 14.4% of children’s and adolescents’ daily energy intake, a figure similar to estimates from 2003 to 2004 (14.1%) and higher than those from 2009 to 2010 (10.6%) [1]. Studies have shown that consumption of food and beverages from fast-food and full-service restaurants among young children and adolescents is associated with higher daily energy intake and poorer diet quality, including higher intake of sugars and sugar-sweetened beverages (SSBs) [2]. A number of studies have documented the low nutritional quality of kids’ meals at fast-food restaurants [[3], [4], [5], [6]], including beverage options that contribute to higher calorie and sugar contents [6]. Further, SSBs are almost universally available at restaurants and overwhelmingly offered with kids’ meals. For example, a study of fast-food and full-service restaurants found that 80% of kids’ meal beverage offerings were SSBs [7]. This is of concern because SSBs are the largest source of added sugars in the American diet [8], and their excessive intake is linked to obesity [9], including among children [10,11].

As a result, many institutions, states, and localities have launched programs or enacted policies to improve the nutritional quality of kids’ meals at restaurants. For example, the National Restaurant Association’s Kids LiveWell program [12] has asked restaurants to voluntarily commit to offering kids’ menu items that meet specific nutritional criteria, including standards for beverages. Several localities have launched similar programs in which restaurants have agreed to modify kids’ menus to offer healthier options and promote them at the point of purchase (e.g., Choose Health LA Restaurants [13]). More than 20 states and localities have enacted legislation that requires restaurants to offer healthier options with kids’ meals. These laws may include requirements for healthy default beverages (i.e., only healthy options are allowable as drinks that automatically come with the kids’ meals), nutritional standards for kids’ meals (e.g., standards for fruits, vegetables, sodium, and sugar), and/or nutritional standards for kids’ meals sold with a toy [[14], [15], [16], [17]].

A number of studies have assessed voluntary programs to improve the nutritional quality of beverages offered with kids’ meals, finding mixed results. A study of the Choose Health LA Restaurants initiative found that of 10 restaurant brands that participated and offered kids’ meals, 6 changed the type of beverages included with the kids’ meals following participation in the program; 5 switched to a healthier beverage, and 1 removed beverages from the meal altogether [18]. An evaluation of the Kids LiveWell program found no difference, over time, in the mean number of calories from beverages on kids’ menus between participating and nonparticipating chain restaurants. Although there was a small reduction in soda offerings across all chain restaurants, it was offset by an increase in flavored milk offerings [7]. In 1 participating Kids LiveWell restaurant chain, an assessment of orders from before to 1 year after participation in the program found a modest decrease in the percentage of kids’ beverage orders for soda (34.7% to 29.7%) and an increase in orders for milk (37.0% to 39.5%) and juice (28.3% to 30.8%) [19]. The changes from baseline increased slightly 2 years after menu changes were implemented [20]. One cross-sectional study found that compared with restaurants that had not pledged to meet voluntary nutritional standards for kids’ meals, restaurants that had pledged were more likely to offer unflavored milk (92.0% compared with 43.2%) and water (60.0% compared with 32.6%) and less likely to offer SSBs (29.1% compared with 38.6%) in kids’ meals on the menu board, although the difference for SSBs was not statistically significant [21]. Another study showed that although fast-food restaurant chains that pledged to offer healthier drinks and sides on kids’ menus fulfilled this commitment on their national menus, their implementation varied widely by individual locations, with many locations listing unhealthy beverages with kids’ meals on menu boards and/or verbally offering such beverages to customers ordering kids’ meals [22].

A limited number of studies have evaluated compliance with government mandates. Similar to findings from evaluations of voluntary pledges or programs, these studies found mixed results related to beverages offered with kids’ meals. A pre-post study of healthy default beverage policies enacted in CA and Wilmington, DE, found that more fast-food restaurants in low-income communities in CA offered healthy beverages as the default option with kids’ meals on the menu board after a state law came into effect (the percentage of restaurants offering healthier beverages complying with the state law increased from 9.7% at baseline to 66.1% after the policy came into effect). There was no change, however, in the proportion of restaurants offering healthy beverage options with kids’ meals in Wilmington (30.8% of restaurants offered healthy beverages with kids’ meals on the menu board at both the time points) [23]. Further, in CA, fewer sales associates offered healthy beverages to customers verbally when a kids’ meal was ordered (the verbal offers decreased from 5.0% at baseline to 1.0% after policy enactment) [23]. Additionally, a cross-sectional study in low-income neighborhoods found that less than half (40.5%) of restaurant-specific and third-party online platforms were compliant with the CA law after enactment [24]. A pre-post intervention-comparison site evaluation of a default beverage policy in Columbus, OH, found no overall change in the default beverages offered with kids’ meals on restaurant websites and third-party online platforms compared with changes in those offered at fast-food restaurants that were not subject to the policy [25].

An IL healthy beverage default (HBD) act, passed in August 2021 and effective from 1 January, 2022, is the most recent state legislation that requires restaurants to offer only healthy beverages with kids’ meals by default (i.e., automatically included, absent requests for an alternative beverage). The IL HBD Act only allows the following beverages as default beverages: *1*) water with no added natural or artificial sweeteners, which may be sparkling or flavored; *2*) 100% juice, which may be diluted with plain or carbonated water, in a serving size of ≤8 oz; *3*) nonfat or 1% dairy milk with ≤130 calories per serving; and *4*) nondairy milk with no added natural or artificial sweeteners, with ≤130 calories per serving, which must meet the standards of the National School Lunch Program [26]. The IL HBD Act differs from similar state laws that have been previously enacted in several ways. For example, the CA HBD law prohibits restaurants from serving any flavored milk or 100% juice by default with kids’ meals, although it does not restrict the fat or calorie content of unflavored milk [16]. An HI law allows 100% juice but does not allow flavored milk [27]. A DE law allows 100% juice and flavored milk with any fat content; however, these drinks are limited to ≤8 oz [28].

This study examined changes in default beverages offered with kids’ meals on the website or application menus of fast-food restaurants following the date on which the IL HBD Act became effective relative to changes on the websites or applications of fast-food restaurants in the comparison site of WI, which does not have an HBD policy. This is the first study, to our knowledge, to evaluate compliance by restaurants with the IL HBD Act and the first to assess the impact of a statewide HBD policy on changes in beverages offered with kids’ meals using a pre-post intervention-comparison site study design.

## Methods

Restaurants in this study were sampled from 12 national fast-food restaurant chains serving kids’ meals with locations in both IL and WI. The 2013 National Center for Health Statistics Urban-Rural Classification Scheme for Counties was used to ensure that restaurants were sampled from both urban and rural areas [29]. Fast-food restaurants were selected from the most populated urban county in IL, Cook, and 2 smaller IL counties, DeKalb (large fringe metro) and LaSalle (micropolitan). In the comparison site of WI, restaurants were sampled from its most populous county, Milwaukee, and 2 smaller counties, Kenosha (large fringe metro) and Walworth (micropolitan).

Before the IL HBD Act became effective on 1 January, 2022, baseline field audits were conducted from 29 October to 18 November, 2021, to assess the beverages offered at the physical locations of the restaurants. Menu data were collected for the same restaurants from 18 November to 24 November, 2021, from online restaurant websites or applications as well as third-party ordering platforms (Grubhub, Uber Eats, and DoorDash). Short-term follow-up data were collected 4 mo after the date on which the policy became effective (5 May to 2 June, 2022) from the websites or applications of the restaurants. Short-term follow-up data were not collected from the physical locations of the restaurants or from third-party ordering platforms.

Data for this study were collected and coded using the Food Policy Program Fast-Food Restaurant Kids’ Meal (FPP-FFKM) audit tool, which provides highly reliable measures of default beverages offered with kids’ meals and their characteristics [30]. The development and testing of the FPP-FFKM tool are described in detail elsewhere [30]. Briefly, trained researchers collected information on the restaurants’ kids' menus, beverage offerings, and nutrient details (e.g., nutrition facts or nutrition labels) needed to determine beverage compliance such as serving size for 100% juice as well as milk fat and calories for dairy milk. When online menus were assessed, the “default” beverage was defined as the first beverage (or beverages) shown to consumers with kids’ meals (compared with “secondary offerings,” which were defined as drinks not visible until the consumer actively requested to see additional beverages). Default beverages were classified into 1 of 18 mutually exclusive beverage categories. Beverage compliance with the IL HBD Act was determined by its category and characteristics.

At baseline, our sample consisted of a total of 201 fast-food restaurants. For this evaluation, 25 restaurants were excluded from 1 chain that had voluntarily committed to providing healthier beverage options with kids’ meals, which was fulfilled consistently across all chain locations. From this sample of 176 restaurants, 39 restaurants did not have a website or application at baseline and were excluded from these analyses. After these exclusions, only 1 location remained for 1 of the chains; so, it was excluded from the analytic sample. Finally, 15 restaurants were excluded because their overall compliance could not be determined at baseline because the type of milk offered by default could not be determined, leaving a final analytic sample of 121 restaurants for this study, which included 64 in IL and 57 in WI, across 8 chains.

Summary statistics were computed on restaurant offerings by site and time period, and difference-in-differences logistic regression models with robust standard errors clustered on the restaurants were computed to examine changes over time in IL compared with those in WI. Summary statistics and regression models were weighted so that each chain would receive the same total weight at each site and time period. Analyses were performed using Stata/SE 13.1 (StataCorp LP).

## Results

Prior to the date on which the IL HBD Act became effective, just over one third of the fast-food restaurants offered bottled plain water (37% in IL and 38% in WI), 88% offered milk, and 75% offered juice as default beverages with kids’ meals on their websites or applications (Table 1). Three quarters (75%) of the restaurants in IL and 68% in WI offered an SSB as a default beverage with kids’ meals, with a similar proportion of restaurants offering soda. Additionally, 15% of the fast-food restaurants in IL and 20% in WI met the criteria of the IL HBD Act for default beverages offered with kids’ meals on their websites or applications.

**Table 1:** Default beverage offerings with kids’ meals in IL and WI before and 4 mo after the effective date of an IL Act requiring healthy beverage defaults^1^

	IL		WI		Difference-in-differences
Beverage offering	Before (%)	After (%)	Before (%)	After (%)	OR (95% CI)
Bottled plain water	37	25	38	25	1.01 (0.57, 1.79)
Milk	88	83	88	75	1.59 (0.85, 2.99)
Compliant^2^	86	78	86	71	1.40 (0.70, 2.82)
Noncompliant^2^	40	29	43	27	1.18 (0.41, 3.38)
Juice	75	67	75	75	0.69 (0.27, 1.73)
Compliant	67	67	75	75	1.00 (0.43, 2.35)
Noncompliant	46	33	50	33	1.22 (0.54, 2.78)
Regular lemonade	25	26	28	25	1.22 (0.90, 1.64)
Soda	75	50	68	50	0.69 (0.32, 1.51)
Sports drinks	27	36	35	38	1.39 (0.73, 2.64)
Energy drinks	0	0	0	0	—
Tea or iced tea	39	22	42	25	0.93 (0.41, 2.16)
Other^3^	41	38	38	38	0.90 (0.45, 1.82)
Any SSB^4^	75	50	68	50	0.69 (0.32, 1.51)
Fountain drink or cup option	41	28	30	25	0.70 (0.31, 1.58)
Overall compliance	15	38	20	39	1.40 (0.45, 4.31)
N	64	64	57	57	

SSB, sugar-sweetened beverage.

1 Values are percentages of restaurants offering each beverage type or complying with the provisions of the IL Act, together with ORs corresponding to difference-in-differences coefficients from logistic regression models with robust standard errors clustered on the restaurant. All statistics are weighted so that each restaurant chain receives equal weight in each site and time point.

2 Two restaurants in IL and 2 in WI were excluded because enough information was not available to assess compliance for milk.

3 Other beverages included artificially sweetened lemonade and juice, unsweetened tea or iced tea, half-tea and half-lemonade mixtures, fountain sparkling water, sparkling water sweetened with juice, Vitamin Water, limeade, slushes, and frozen drinks.

4 Includes any beverage with added sugars, except milk.

There was no statistically significant increase in compliance with the IL HBD Act criteria on the websites or applications of the fast-food restaurants 4 mo after the date on which the policy became effective in IL relative to that in the comparison site of WI. Although the compliance by the fast-food restaurants increased in IL from 15% to 38% after the policy came into effect, there was a similar pattern in WI (an increase from 20% to 39%). There were no statistically significant changes in the types of compliant beverages offered by default with kids’ meals in IL compared with those in WI.

Table 2 shows the percentage of restaurants that offered only 1 beverage option on their online ordering platform as the default beverage offered with kids’ meals and the percentage of restaurants in compliance with the IL HBD Act broken down by chain (anonymized). Prior to the date on which the IL HBD Act became effective, almost all the restaurants offered multiple default beverages from which to choose (i.e., for most chains, 0% of restaurants offered just a single default beverage option). Following the date on which the IL HBD Act became effective, all restaurants in 2 chains made changes to their online ordering platform to offer only 1 beverage option as the default beverage (either milk or 100% juice, depending on the location). This single beverage offering was compliant across all restaurant locations of these chains in both IL and WI.

**Table 2:** Percentage of restaurants in IL and WI in compliance with an IL Act requiring healthy beverage defaults before and 4 mo after the policy effective date and percentage offering only 1 default beverage option, by chain^1^

	Percent in compliance				Percent offering only 1 option			
	IL		WI		IL		WI	
Chain	Before (%)	After (%)	Before (%)	After (%)	Before (%)	After (%)	Before (%)	After (%)
Chain 1 (*n* = 13, 13, 20, 20)	0	100	0	100	0	100	0	100
Chain 2 (*n* = 11, 11, 5, 5)	0	0	60	0	0	0	0	0
Chain 3 (*n* = 12, 12, 11, 11)	0	100	0	100	0	100	0	100
Chain 4 (*n* = 3, 3, 2, 2)	100	100	100	100	0	0	0	0
Chain 5 (*n* = 2, 2, 2, 2)	0	0	0	0	0	0	0	0
Chain 6 (*n* = 6, 6, 11, 11)	17	0	0	9	0	0	0	0
Chain 7 (*n* = 10, 10, 5, 5)	0	0	0	0	10	0	0	0
Chain 8 (*n* = 7, 7, 1, 1)	0	0	0	0	0	0	0	0

1 Sample sizes by site and time period are shown at the beginning of each row separated by commas.

## Discussion

The results of this evaluation showed that 4 mo after the IL HBD Act came into effect, there was no increase in compliance of default beverages offered with kids’ meals on the websites or applications of fast-food restaurants in IL compared with the comparison restaurants in WI. Although the compliance increased in IL, it similarly increased in WI, and, thus, there were no significant changes in offerings in IL that could be directly attributed to the IL HBD Act. Further examination revealed that restaurants from 2 fast-food chains made changes to their online ordering platforms in both the states, reducing the number of default beverages offered with kids’ meals to just a single compliant offering.

To date, only 1 other study has assessed the extent to which the online ordering platforms of fast-food restaurants comply with a state-level HBD policy; however, that study included neither prepolicy data nor comparison restaurants that were not subject to the policy, making it difficult to distinguish the effects of the policy from voluntary restaurant actions or pre-existing trends. That study evaluated the CA HBD policy and found that 40.5% of online platforms of fast-food restaurants (38.5% of restaurant-specific platforms and 41.7% of third-party platforms) complied with the policy approximately 2 years after it came into effect [24]. Our study similarly showed low compliance, with 38% of websites or applications of fast-food restaurants in compliance with the IL HBD Act 4 mo after the date on which it became effective; and, no changes were attributable to the IL HBD policy. The limited research assessing the physical locations of restaurants showed mixed results for compliance. One CA study found that the proportion of restaurants offering compliant beverages on their onsite menu boards increased significantly after the CA HBD law took effect, although comparison restaurants were not included in the study [23]. A study of Wilmington, DE, found no changes in the proportion of restaurants offering compliant beverages after an HBD policy came into effect; however, the study sample size was small (*n* = 14) [23]. There may be differences in compliance by restaurants online compared with that in restaurant because the authority of the regulatory agency to enforce online activity is less certain, both under the policy and its statutory mandate [31].

This study is subject to several limitations. First, we only collected 4-mo follow-up data from menus on the websites or applications of the restaurants; so, investigations of short-term effects of the policy on other menus, such as interior menu boards, were not performed. However, long-term follow-up data (10 mo after the date on which it became effective) will include all platforms, including interior menu boards and electronic kiosks, drive-through menus, restaurant websites or applications, and third-party online platforms. Second, this analysis focused on compliance and did not collect data on orders or purchases of kids’ meals; so, it could not assess changes in the intake of beverages by children from restaurants; however, changes cannot be expected without resultant changes in offerings. Third, identification of differences between intervention and comparison sites may be difficult if the online menu offerings of chain fast-food restaurants are regionally or nationally coordinated. Fourth, we only assessed menus from chain fast-food restaurants, and compliance may be appreciably different in full-service or independently owned restaurants. Lastly, it is important to note that this study presented short-term results collected during the COVID-19 pandemic. It is unclear as to what extent this policy was implemented and enforced by state and local health agencies at the time of follow-up data collection, which came in the wake of relatively recent closures of restaurants, suspended restaurant inspections, and competing health agency priorities. The Act specifies that compliance will be assessed during health department inspections and that those in violation will be subject to a warning for the first offense, a fine of $25 for the second offense, and a fine of $100 for any subsequent offenses [26]. With additional time and resources to communicate the HBD policy to restaurants, provide technical assistance, and consistently issue citations for violations, policy compliance may improve.

Additionally, it is important to note that our study of the IL HBD Act and a previous study of the policy in Columbus, OH, which similarly found no changes in default beverages offered with kids’ meals relative to changes in restaurants not subject to the default beverage restrictions, were both conducted only 4 mo after the date on which the policy became effective. Implementation by restaurants may require more time, and guidance by government agencies may be needed regarding whether and which types of online platforms are subject to policy requirements. Indeed, the results of this study provide further evidence that highlights the need for early technical assistance with clear communication of the required changes and enforcement to ensure that restaurants make changes broadly, including on online platforms, and that implementation is operationalized without substantial lags.

Overall, given the results of this study and the mixed findings from previous studies that employed various methods that prohibit the identification of policy effects independent of other trends, continued evaluation of policy impacts using pre-post intervention-comparison study designs will be important, as will investigations across all types of platforms from which customers can order kids’ meals from restaurants. Future studies should continue to measure the effectiveness of HBD policies alongside implementation strategies to determine how these policies can best achieve the desired improvements in the nutritional quality of kids’ meals at restaurants.

## Author Contributions

The authors’ responsibilities were as follow – LMP: designed the research; LMP, JL, AAP, and AM: developed the audit tool; AAP and JL: managed and conducted the research; JL: performed statistical analysis; JL and LMP: analyzed the data; LMP, JL, and AM: wrote the paper; LMP: was primarily responsible for the final content; and all authors: read and approved the final manuscript.

## Funding

Supported by a grant from the Bloomberg Philanthropies’ Food Policy Program (grant #2020-85774). The contents of this publication do not necessarily reflect the views or policies of Bloomberg Philanthropies. Access to the REDCap data system was provided by the University of Illinois Chicago Center for Clinical and Translational Science (grant #UL1TR002003). Neither of these organizations was involved in study design, collection, analysis, and interpretation of data, writing of the report, or any restrictions regarding the submission of the report for publication.

## Author disclosures

The authors report no conflicts of interest.

## Data availability

Data described in the manuscript, code book, and analytic code will be made available upon request pending application.

## References

[1] Fryar C.D., Carroll M.D., Ahluwalia N., Ogden C.L. (2020). Fast food intake among children and adolescents in the United States, 2015-2018. NCHS Data Brief.

[2] Powell L.M., Nguyen B.T. (2013). Fast-food and full-service restaurant consumption among children and adolescents: effect on energy, beverage, and nutrient intake. JAMA Pediatr.

[3] Batada A., Bruening M., Marchlewicz E.H., Story M., Wootan M.G. (2012). Poor nutrition on the menu: children’s meals at America’s top chain restaurants. Child Obes.

[4] Deierlein A.L., Peat K., Claudio L. (2015). Comparison of the nutrient content of children’s menu items at US restaurant chains, 2010–2014. Nutr. J..

[5] Sliwa S., Anzman-Frasca S., Lynskey V., Washburn K., Economos C. (2016). Assessing the availability of healthier children’s meals at leading quick-service and full-service restaurants. J. Nutr. Educ. Behav..

[6] Dunn C.G., Vercammen K.A., Frelier J.M., Moran A.J., Bleich S.N. (2020). Nutrition composition of children’s meals in twenty-six large US chain restaurants. Public Health Nutr.

[7] Moran A.J., Block J.P., Goshev S.G., Bleich S.N., Roberto C.A. (2017). Trends in nutrient content of children’s menu items in U.S. chain restaurants. Am. J. Prev. Med..

[8] US Department of Agriculture, US Department of Health and Human Services (2020). https://www.dietaryguidelines.gov/resources/2020-2025-dietary-guidelines-online-materials.

[9] Malik V.S., Hu F.B. (2019). Sugar-sweetened beverages and cardiometabolic health: an update of the evidence. Nutrients.

[10] Bleich S.N., Vercammen K.A. (2018). The negative impact of sugar-sweetened beverages on children’s health: an update of the literature. BMC Obes.

[11] Bowman S.A., Clemens J.C., Friday J.E., Schroeder N., LaComb R.P. (2019). Added sugars in American children’s diet: what we eat in America, NHANES 2015-2016. Dietary Data Brief No. 26, Food Surveys Research Group (USDA-ARS). [Internet].

[12] (2022). National Restaurant Association. Kids LiveWell. [Internet].

[13] County of Los Angeles Public Health (2022). http://publichealth.lacounty.gov/chronic/restaurants/restaurantshome.htm.

[14] OH Columbus (2020). https://columbus.legistar.com/LegislationDetail.aspx?ID=4711383&GUID=B2BC5267-18D1-491B-A4C6-54A490E5268F&Options=ID%7C&Search=2870-2020&FullText=1.

[15] DE Wilmington (2018). https://www.wilmingtoncitycouncil.com/wp-content/uploads/2018/09/Ord.-18-046-4576-Rev.-1-Amend-Ch.-5-Healthy-Childrens-Meal-100418.pdf#:%7E:text=SYNOPSIS%3A%20This%20Ordinance%20amends%20Chapter%205%20of%20the,or%20alternative%20beverage%20if%20requested%20by%20the%20customer.

[16] (2018). State of California. Senate bill no. 1192. An act to add chapter 12.8 (commencing with section 114379) to part 7 of division 104 of the health and safety code, relating to children’s health. [Internet].

[17] Perez C.L., Moran A.J., Headrick G., McCarthy J., Cradock A.L., Pollack Porter K.M. (2022). State and local healthy kids’ meal laws in the United States: a review and content analysis. J. Acad. Nutr. Diet..

[18] Gase L.N., Kaur M., Dunning L., Montes C., Kuo T. (2015). What menu changes do restaurants make after joining a voluntary restaurant recognition program?. Appetite.

[19] Anzman-Frasca S., Mueller M.P., Sliwa S., Dolan P.R., Harelick L., Roberts S.B. (2015). Changes in children’s meal orders following healthy menu modifications at a regional US restaurant chain. Obes..

[20] Anzman-Frasca S., Mueller M.P., Lynskey V.M., Harelick L., Economos C.D. (2015). Orders of healthier children’s items remain high more than two years after menu changes at a regional restaurant chain. Health Aff.

[21] Harpainter P., Hewawitharana S.C., Lee D.L., Martin A.C., Gosliner W., Ritchie L.D. (2020). Voluntary kids’ meal beverage standards: are they sufficient to ensure healthier restaurant practices and consumer choices?. Int. J. Environ. Res. Public Health..

[22] Harris J., Hyary M., Seymour N., Choi Y.-Y. (2017). Are fast-food restaurants keeping their promises to offer healthier kids' meals? Hartford, CT: University of Connecticut. Rudd Center for Food Policy and Obesity. [Internet].

[23] Ritchie L.D., Lessard L., Harpainter P., Tsai M.M., Woodward-Lopez G., Tracy T. (2022). Restaurant kids’ meal beverage offerings before and after implementation of healthy default beverage policy statewide in California compared with citywide in Wilmington, Delaware. Public Health Nutr.

[24] Thompson H.R., Martin A., Strochlic R., Singh S., Woodward-Lopez G. (2022). Limited implementation of California's healthy default beverage law for children's meals sold online. Public Health Nutr.

[25] Pipito A.A., Beal V.G., Leider J., Powell L.M. (2022). Research brief no. 126.

[26] (2021). State of Illinois. Public act 102-0681 amend. § 21.5. Default beverage for children’s meals. [Internet].

[27] (2020). HI Rev Stat §321-30.3 Default beverages offered with children’s meals. [Internet].

[28] Del. Laws c. (2019). 100, § 2 amend. § 140B Default beverages offered in children's meals. [Internet].

[29] (2017). National Center for Health Statistics (NCHS), Centers for Disease Control and Prevention. NCHS urban-rural classification scheme for counties. [Internet].

[30] Powell L.M., Leider J., Pipito A.A., Marinello S., Szkorla A., Moran A. (2022).

[31] Pomeranz J., Cash S., Springer M., Del Giudice I., Mozaffarian D. (2022). Opportunities to address the failure of online food retailers to ensure access to required food labelling information in the USA. Public Health Nutr.

